# Induction of Malignant Plasma Cell Proliferation by Eosinophils

**DOI:** 10.1371/journal.pone.0070554

**Published:** 2013-07-22

**Authors:** Tina W. Wong, Hirohito Kita, Curtis A. Hanson, Denise K. Walters, Bonnie K. Arendt, Diane F. Jelinek

**Affiliations:** 1 Department of Immunology, Mayo Clinic, Rochester, Minneapolis, United States of America; 2 Department of Internal Medicine, Mayo Clinic, Rochester, Minneapolis, United States of America; 3 Department of Laboratory Medicine and Pathology, Mayo Clinic, Rochester, Minneapolis, United States of America; Centro di Riferimento Oncologico, IRCCS National Cancer Institute, Italy

## Abstract

The biology of the malignant plasma cells (PCs) in multiple myeloma (MM) is highly influenced by the bone marrow (BM) microenvironment in which they reside. More specifically, BM stromal cells (SCs) are known to interact with MM cells to promote MM cell survival and proliferation. By contrast, it is unclear if innate immune cells within this same space also actively participate in the pathology of MM. Our study shows for the first time that eosinophils (Eos) can contribute to the biology of MM by enhancing the proliferation of some malignant PCs. We first demonstrate that PCs and Eos can be found in close proximity in the BM. In culture, Eos were found to augment MM cell proliferation that is predominantly mediated through a soluble factor(s). Fractionation of cell-free supernatants and neutralization studies demonstrated that this activity is independent of Eos-derived microparticles and a proliferation-inducing ligand (APRIL), respectively. Using a multicellular *in vitro* system designed to resemble the native MM niche, SCs and Eos were shown to have non-redundant roles in their support of MM cell growth. Whereas SCs induce MM cell proliferation predominantly through the secretion of IL-6, Eos stimulate growth of these malignant cells via an IL-6-independent mechanism. Taken together, our study demonstrates for the first time a role for Eos in the pathology of MM and suggests that therapeutic strategies targeting these cells may be beneficial.

## Introduction

Multiple myeloma (MM) is a plasma cell (PC) malignancy that accounts for 10% of all hematologic malignancies in the United States. Over 20,000 new cases of MM are diagnosed each year in the US making it the second most common hematologic malignancy after non-Hodgkin lymphoma.[1] Clinically, MM is differentiated from its premalignant form, monoclonal gammopathy of undetermined significance (MGUS), and smoldering multiple myeloma (SMM), by the abundance (>10%) of clonal PCs in the bone marrow (BM), a serum monoclonal immunoglobulin M protein of >3 g/dl, and the presence of end organ damage that includes hypercalcemia, renal insufficiency, anemia, and lytic bone lesions.[2] Even though numerous therapeutic options exist for the treatment of MM and that the median overall survival for patients with MM has more than doubled from 3 to 7 years over the last decade as a result of novel drugs, the disease remains incurable.[3], [4] A greater understanding of the biology of MM will facilitate design of improved therapeutic strategies.

Similar to many other cancers, MM cells can harbor a number of genetic abnormalities, including chromosomal translocations, hyperdiploidy, and gene-specific mutations.[2] Interestingly, most of these genetic changes are also present in the pre-malignant MGUS stage. Given this, we believe other factors within the tumor microenvironment must contribute to disease progression by influencing cell survival and/or proliferation.

The BM microenvironment in which MM cells reside is made up of cellular and noncellular compartments. The cellular compartment is comprised of hematopoietic cells as well as nonhematopoietic cells such as osteoclasts, osteoblasts, endothelial cells, and stromal cells (SCs). The noncellular compartment consists of a structural unit made by extracellular matrix together with a mixture of chemokines, cytokines, and growth factors. Both compartments have been shown to interact with MM cells and contribute toward tumor growth and disease pathology.[5], [6] Interleukin-6 (IL-6), vascular endothelial growth factor (VEGF), and insulin-like growth factor 1 are secreted by BM SCs, osteoclasts, osteoblasts, and/or MM cells themselves and each of these soluble factors stimulates MM cell growth and/or survival. Additionally, VEGF can induce neovascularization in order for tumor cells to receive an adequate supply of oxygen and nutrients. The chemokine CXCL12, while being able to direct homing of MM cells to the BM, has also been shown to exhibit proliferation-inducing effects on MM cells.[7] The intercommunication between MM cells, SCs, osteoclasts, and osteoblasts through factors such as receptor activator of nuclear factor-κB ligand, macrophage inflammatory protein-1α, dickkopf-1, monocyte chemotactic protein-1 (MCP-1), and interleukin 3 (IL-3) have been demonstrated to influence bone resorption by osteoclasts and bone formation by osteoblasts thus leading to osteolytic bone lesions often seen in this disease.

The role of non-lymphocyte hematopoietic cells in MM has been much less well characterized. Although a number of studies have focused on the role of macrophages, megakaryocytes, basophils, dendritic cells, and most recently eosinophils (Eos) in the maintenance of normal BM PC homeostasis,[8], [9], [10], [11], [12], [13] not much is known regarding their interactions with malignant PCs. Of the above listed cell types, macrophages and dendritic cells are the only innate immune cells that have been demonstrated to influence MM cell growth to date.[14], [15]

As mentioned, Eos were recently demonstrated to play a role in the maintenance of normal BM PC longevity.[13] Using transgenic mice engineered to be deficient in Eos, Chu et al demonstrated that PC survival in the BM at baseline and after immunization was dependent on the presence of Eos. Reconstitution of these Eos-deficient transgenic mice with Eos rescued the retention of PCs in the BM. In a subsequent study, it was demonstrated that activation of Eos leads to an enhanced production of PC survival factors including APRIL, IL-6, IL-4, IL-10, and tumor necrosis factor-α by these cells.[16] However, as these studies utilized mouse models to study the Eos-PC interaction in normal immune responses, it remains unknown if Eos are also required for the long term survival of PCs in humans. Furthermore, given the ability of Eos to affect normal BM PC biology, we question whether Eos may be involved in the pathophysiology of malignant PCs. Thus, in this study we examined the possibility that MM cells may hijack this interaction with Eos in order to gain a proliferative advantage.

## Materials and Methods

### Ethical statement and patient cohort

Mayo Clinic Institutional Review Board (IRB) approval was obtained for the study of normal and malignant plasma cell and the bone marrow microenvironment. Mayo Clinic IRB approved the acquirement of BM aspirates from patients undergoing spine surgeries without coincident B-lineage malignancies as well as from patients with monoclonal gammopathies during routine clinical examination. BM core biopsies were obtained from monoclonal gammopathy patients as well as patients with no evident marrow/hematologic malignancies. BM tissues were collected and used only from patients providing written informed consent in accord with Helsinki protocol. Regarding peripheral blood (PB) samples, Mayo Clinic IRB approval was obtained and blood was drawn from patients with monoclonal gammopathies as part of the clinical examination or from healthy, non-smoking individuals who have no clinical history of immunological disorders. All blood specimens were collected only after written informed consent by the patients in accordance with the Declaration of Helsinki.

### Cell lines and culture medium

The human myeloma cell lines (HMCLs) used in this study were all derived in our laboratory, which includes KAS-6/1, DP-6, KP-6, JMW, ALMC-2, and ANBL-6. [17], [18], [19] Each of these HMCLs were established from patients who provided their written informed consent as described above. The human BM stromal cell line, HS-5, was purchased from ATCC (Manassas, VA, USA). All HMCLs were maintained in Iscove's Modified Dulbecco's Medium (IMDM; Gibco Life Technologies, Grand Island, NY, USA) supplemented with 100 U/ml penicillin G, 10 µg/ml streptomycin (Invitrogen, Carlsbad, CA, USA), 50 µg/ml gentamycin (Gibco Life Technologies), 1 ng/ml recombinant IL-6 (Novartis, Basel, Switzerland), and 5% heat-inactivated fetal calf serum (FCS) (PAA Laboratories, Etobicoke, Ontario, Canada). HS-5 cells were maintained in Dulbecco's Modified Eagle Medium (Gibco Life Technologies) supplemented with 100 U/ml penicillin G, 10 µg/ml streptomycin, 3 µg/ml L-glutamine (Invitrogen), 50 µg/ml gentamycin, and 10% heat-inactivated FCS.

### Isolation of primary MM cells

Mononuclear cells were isolated from BM aspirates of MM patients via Ficoll-Paque (GE Healthcare, Piscataway, NJ, USA) density centrifugation. Primary MM cells were subsequently isolated via magnetic bead separation using human CD138 positive selection kits (StemCell Technologies, Vancouver, British Columbia, Canada) and an automated Robosep Cell Separator (StemCell Technologies) according to the manufacturer's protocol.

### Isolation of PB and BM Eos

PB Eos were isolated using anti-CD16-conjugated magnetic beads (Miltenyi Biotec, Auburn, CA, USA) following the manufacturer's protocol. Briefly, blood granulocytes were recovered from whole blood via Percoll density centrifugation. Subsequent incubation of these granulocytes with anti-CD16-conjugated magnetic beads for negative selection led to the retention of CD16^+^ neutrophils in a MACS column (Miltenyi Biotec) and recovery of untouched, CD16^−^ PB eosinophils. BM Eos were purified from aspirates as described previously.[20] Briefly, granulocytes were isolated from the BM via Percoll density centrifugation followed by an *in vitro* 8-day culture in a pro-Eos-survival medium (RPMI_eos_) containing 1 ng/ml recombinant IL-5 (Peprotech, Rocky Hill, NJ, USA).

### Histology and immunofluorescence microscopy

Formalin-fixed, paraffin-embedded BM biopsy samples were sectioned (3.0 µm thickness) onto glass slides and baked at 60°C for 2 hr. De-paraffinization was achieved in 1% iodine/xylene baths followed by incremental alcohol rehydration steps. Slides were either stained with hematoxylin and eosin (H&E) or heated for 10 minutes in 1 mM EDTA buffer pH 8.0 for antigen/epitope retrieval for immunofluorescence staining. PCs were stained with unconjugated CD138 antibody (DAKO, Denmark) and fluorescein-conjugated goat anti-mouse Ig (Biosource, Carlsbad, CA, USA). Eos were stained with 1% Chromotrope 2R (Sigma Aldrich, St Louis, MO, USA). Stained slides were coverslipped with Vectashield mounting medium (Vector Labs, Burlingame, CA, USA). H&E stained sections were viewed on an Olympus Provis AX70 Upright Compound Microscope (Olympus, Center Valley, PA, USA) and images were taken using an Olympus DP71 camera (Olympus). Immunofluorescence images were obtained using a LSM 780 Confocal Laser Scanning Microscope (Carl Zeiss MicroImaging, Inc., Oberkochen, Germany). Quantitation of PC and Eos proximity was performed on immunofluorescence-stained sections by scoring 6 random medium-power (40× objective) fields for each patient sample and quantitating as follows: 1) Eos in direct contact with PCs; 2) Eos within a 3-cell distance of PCs; and 3) Eos more than a 3-cell distance away from the closest PC. Images were obtained and Eos-PC proximity was scored blinded to the patients' clinical diagnoses.

### Proliferation assays

Proliferation of HMCLs was determined using DNA-synthesis assays as measured by [^3^H]-thymidine (PerkinElmer, Waltham, MA, USA) incorporation. Cells were incubated in IMDM containing 5% FCS with or without IL-6 (1 ng/ml) at 37°C in 5% CO_2_ for 3 days prior to analysis of DNA synthesis. For co-cultures of HMCL and Eos, 1 ng/ml IL-5 was added for the maintenance of Eos survival and cells were plated at a 1∶1 ratio unless otherwise indicated. In co-cultures of HMCL and HS-5, cells were plated at a 4∶1 ratio unless otherwise indicated. Prior to plating, HS-5 were partially irradiated with 1730 rads to inhibit their proliferation. 24 hr Eos and HS-5 culture supernatants (SN) were collected from purified PB/BM Eos and HS-5 cultured at 3 and 0.6×10^6^ cells/ml, respectively, in RPMI_eos_ and used to treat HMCLs in proliferation assays in a 1∶4 dilution. A two-step centrifugation at 185×g for 10 min followed by 1000×g for 20 min was used to remove cells and cell debris from culture SNs. Control medium (CM) in these studies was medium used for the SN collection (i.e., RPMI_eos_) incubated at 37°C for the same duration in the absence of any cells. SN and CM were stored at −20°C in 1 ml aliquots until time of use in experiments. 0.4 µm pore transwells (Corning Inc., Corning, NY, USA) were used to assess contact dependency by plating HMCLs in the bottom chamber and Eos within the transwells. For APRIL neutralization studies, 2 µg/ml recombinant human TACI-Ig or control-Ig (R&D, Minneapolis, MN, USA) were preincubated with Eos SN or CM for 2 hr at 37°C prior to addition of HMCLs. For IL-6 blocking studies, 2 µg/ml neutralizing antibodies against IL-6 or isotype control antibodies (R&D) were preincubated with Eos, Eos SN, HS-5 SN, and media containing recombinant IL-6 for 4 hr at 37°C prior to addition to HMCLs. Data are presented as the mean [^3^H]-thymidine incorporation of triplicate samples +/− standard error of the mean (s.e.m.).

Proliferation of primary MM cells was assessed using bromodeoxyuridine (BrdU)-labeling techniques. Isolated primary MM cells were cultured in control media or Eos SN at a 1∶2 dilution in IMDM containing 5% FCS. A BrdU-APC flow kit (BD Biosciences, San Diego, CA, USA) was used; 10 µM BrdU was added to the culture 16 hr prior to staining on day 3 of culture according to the manufacturer's protocol.

### Microvesicle and exosome isolation

Eos SN was initially centrifuged at 2,000×g for 20 min to remove cell debris. Microvesicles (MV) were then isolated from the debris-free Eos SN via ultracentrifugation for 60 min at 17,000×g using an Accuspin Micro 17 Centrifuge (Fischer Scientific, Waltham, MA, USA). The MV-containing pellet was resuspended in 1/3 of the starting volume for HMCL stimulation while a portion of the remaining MV-free supernatant (designated as fraction S1) was further subjected to ultracentrifugation for 60 min at 80,000×g using a TLX Beckman Optima Ultracentrifuge (Beckman Instruments, Palo Alto, CA, USA). The exosome-containing pellet was resuspended in 1/3 of the starting volume and used to stimulate HMCLs along with stimulating with the remaining MV-free, exosome-free supernatant (designated as fraction S2).

### Polymerase chain reaction

Total RNA was extracted from cells using Trizol reagent (Invitrogen) according to the manufacturer's protocol. An iScript cDNA synthesis kit (Bio-Rad, Hercules, CA, USA) was used to generate cDNA. PCR was performed in a conventional 25 µl PCR reaction assay using the Qiagen HotStarTaq MasterMix kit (Qiagen, Valencia, CA, USA). The following primers were used: IL-6, 5′-GGAGACTTGCCTGGTGAAAATC (forward) and 5′-GCTGCGCAGAATGAGATGAGTTG (reverse); beta actin, 5′-GGATCCGACTTCGAGCAAGAGATGGCCAC (forward) and 5′-CAATGCCAGGGTACATGGTGGTG (reverse). The sizes of the PCR products were: IL-6, 268 bp; and beta actin, 271 bp. PCR amplifications were carried out in a Perkin Elmer 9600 thermocycler. A 1.5% TAE agarose gel containing 200 ng/ml ethidium bromide was used to separate PCR products by size.

### Cytokine array

Eos SN was collected from purified BM Eos after culture for 24 hr at 3×10^6^ cells/ml in IMDM containing 10% FCS. Cytokines secreted into the culture SN by Eos were measured using the Human Cytokine Array C3 (RayBiotech, Norcross, GA, USA) and following the manufacturer's protocol. A control array was performed with IMDM containing 10% FCS and 1 ng/ml recombinant IL-5 to simultaneously check for cross-reactivity of the array to fetal calf serum proteins and assess the sensitivity of this method of cytokine detection.

## Results

### Proximity of Eos and PCs in human bone marrow

**Figure 1:**
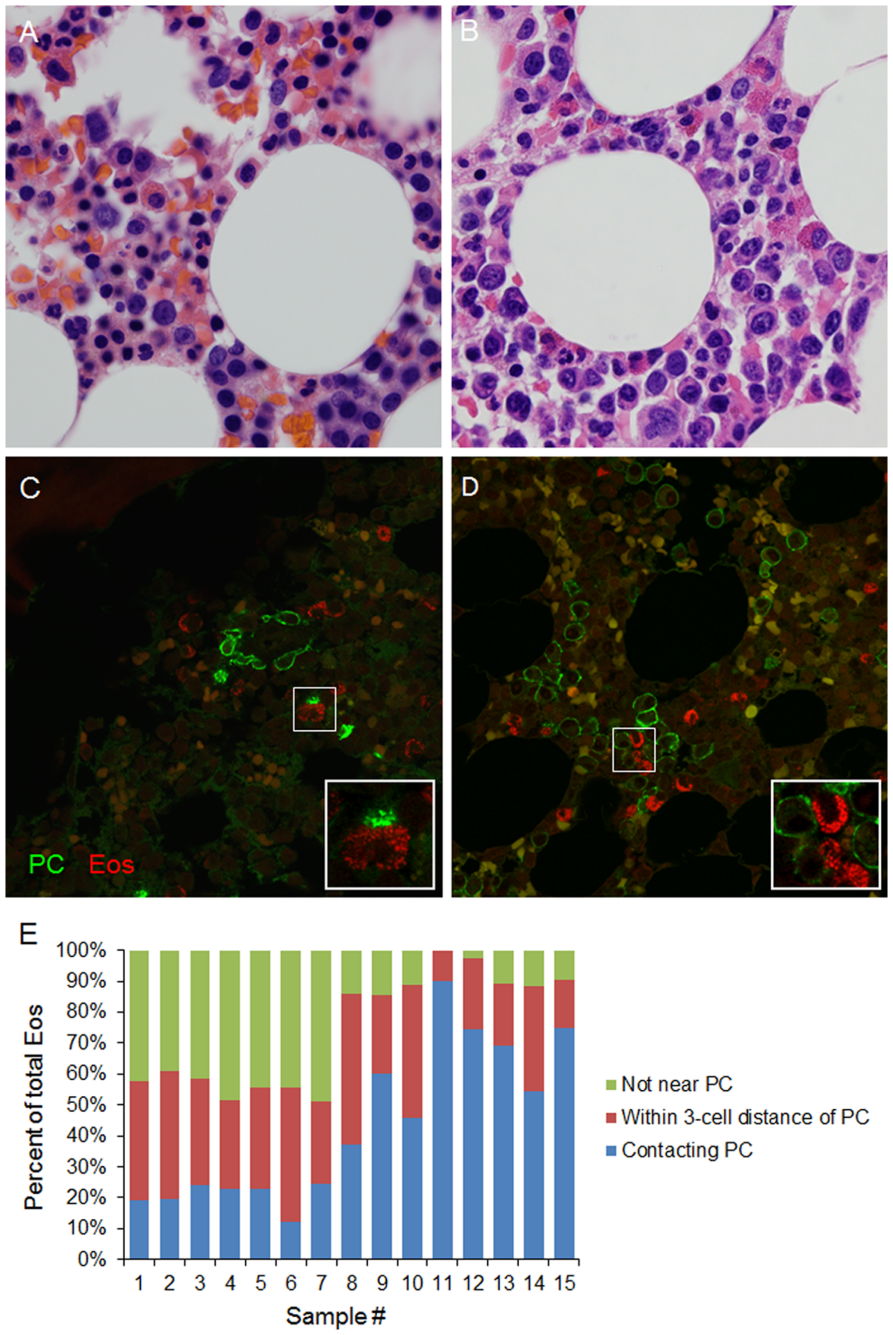
Eosinophils and PCs are found in close proximity in the human bone marrow. BM biopsies were obtained from normal donors (**A, C**) and MM patients (**B, D**) and stained with H&E (**A, B**) or using immunofluorescence (**C, D**) for selective visualization of PCs and Eos. In immunofluorescence staining, PCs were stained with anti-CD138 mAb (green) and Eos were stained with chromotrope 2R (red). Autofluorescent red blood cells are shown in yellow in these overlaid images. Images are representative of 5 normal donor and 10 MM patient BM biopsies. (**E**) Quantitation of Eos across 6 random fields from each immunofluorescence-stained sample dividing Eos into 3 categories: 1) Eos in direct contact with PCs; 2) Eos within a 3-cell distance of PCs; and 3) Eos more than a 3-cell distance away from the closest PC. Samples #1-5 are BM biopsies from normal donors. Samples #6–8 are from patients with MGUS. Samples #10–12 are from patients with SMM. Samples #9 and 13–15 are from patients with MM. See Table S1 for more detail.

We began our studies by assessing whether Eos colocalize with PCs in human bone marrow. BM core biopsies from 5 normal subjects and 10 patients with various stages of monoclonal gammopathy, including MGUS, SMM, and MM, were analyzed. Initial evaluation of the H&E stained biopsies revealed occasional juxtaposition of Eos with PCs in both healthy and MM BMs (Figure 1A and 1B). For the selective visualization of Eos and PCs in these tissues, we implemented immunofluorescence staining and confirmed the proximity of these cell types within the BM (Figure 1C and 1D) using a quantitation strategy described in the Materials and Methods section. This analysis of the biopsy sections revealed increasing percentages of Eos in close proximity with PCs with disease progression (Figure 1E and Table S1).

### Eosinophils enhance proliferation of malignant PCs in vitro

**Figure 2:**
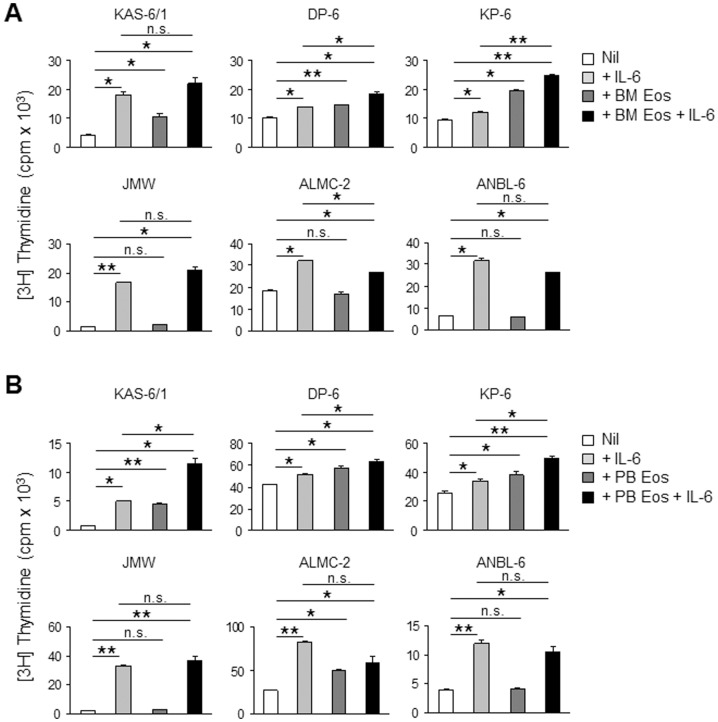
Co-culture of KAS-6/1, DP-6, and KP-6 with Eos enhances HMCL proliferation. HMCL were cultured with Eos isolated from BM (**A**) or PB (**B**) in the presence or absence of IL-6. HMCL and Eos were plated at a 1∶1 ratio. Proliferation of HMCL was assessed by [3H]TdR-incorporation. Results are representative of 3 independent experiments. * *p*<0.05; ** *p*<0.001; n.s., not significant.

To determine whether the presence of Eos influences the biological activity of malignant PCs, we utilized our panel of HMCLs to assess proliferation levels in the absence or presence of Eos. Given that coculture with Eos requires the addition of IL-5, an Eos survival factor, we first verified that the proliferation of these HMCLs was not affected by IL-5 (Figure S1). Additionally, we also tested the effect of IL-5 on HMCL proliferation in the presence of IL-6, a known growth cytokine for myeloma cells, to rule out any possible synergism between these 2 molecules as this has previously been observed for some MM cell lines.[21] However, the data shown in Figure S1 demonstrate that the proliferation of IL-6-stimulated MM cells is not affected by IL-5. To determine whether Eos may induce proliferation of malignant PCs, we tested the HMCLs' proliferation in coculture with BM Eos either in the absence or presence of saturating levels (1 ng/ml) of exogenous IL-6. Our data revealed that BM Eos enhanced the proliferation of KAS-6/1, DP-6, and KP-6 cells but not of JMW, ALMC-2, and ANBL-6 cells (Figure 2A). Additionally, we noted that the proliferation-inducing effect of BM Eos on KAS-6/1, DP-6, and KP-6 cells was not masked by the addition of saturating levels of IL-6. The finding that the coculture of KAS-6/1, DP-6, and KP-6 with BM Eos in the presence of IL-6 induced a greater proliferative response in these HMCLs than did the coculture or the IL-6 treatment alone suggested that BM Eos and IL-6 may act differently and in concert to provide the optimal microenvironment for these malignant PCs in MM.

In a previous study we have compared human Eos isolated from the BM to those isolated from the PB to show similar functionality of these populations of Eos in their ability to be activated upon stimulation with PMA or high dose IL-5.[20] Here we questioned whether Eos isolated from the two different compartments (PB vs BM) have intrinsic differences at baseline that may affect their ability to promote HMCL proliferation. However, our results demonstrate that PB Eos can similarly enhance proliferation of KAS-6/1, DP-6, and KP-6 and not of JMW, ALMC-2, and ANBL-6 cells (Figure 2B).

### Stimulation of HMCL proliferation by Eos is largely mediated by soluble factors

**Figure 3:**
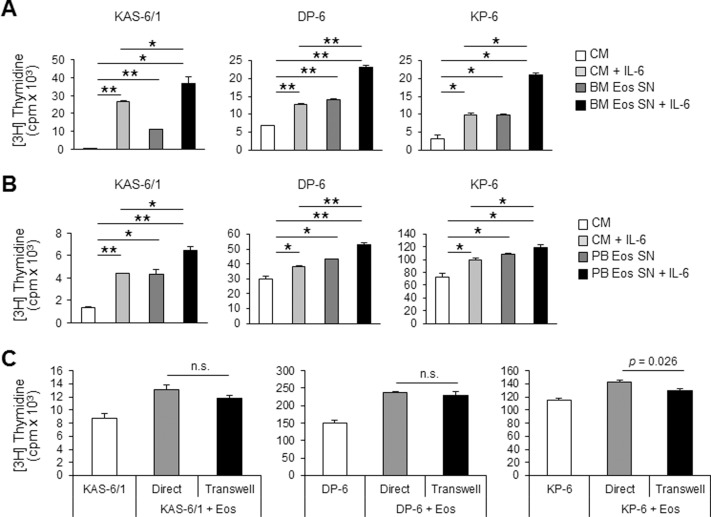
Eos enhance KAS-6/1, DP-6, and KP-6 proliferation in a contact-independent manner. BM and PB Eos SN were collected as described in the Methods section. HMCL were cultured with or without BM (**A**) or PB (**B**) Eos SN in the presence or absence of IL-6. Proliferation of HMCLs was determined via [3H]TdR-incorporation. CM, control medium. (**C**) Proliferation of HMCLs either alone, in direct co-culture with BM Eos, or in the presence of BM Eos across a 0.4 µm-transwell was assessed. Results are representative of 3 independent experiments. * *p*<0.05; ** *p*<0.001; n.s., not significant.

To assess the contact-dependency of Eos-induced proliferation of KAS-6/1, DP-6, and KP-6 cells, we collected cell-free culture SN from BM or PB Eos and evaluated proliferation levels of KAS-6/1, DP-6, and KP-6 cells when cultured with Eos SN in the absence or presence of IL-6. As seen with the co-culture experiments, the treatment of these cell lines with BM or PB Eos SN resulted in enhanced proliferation (Figure 3A and 3B), suggesting the presence of at least a contact-independent component. Similarly, co-culture of Eos and these HMCLs across a 0.4 µm-pore transwell resulted in an increase in HMCL proliferation and confirmed the existence of a contact-independent mechanism (data not shown). Given the ability of Eos SN to enhance HMCL proliferation and because Eos have previously been shown to play a role in the survival of normal long-lived PCs in mice,[13], we also investigated whether there was an effect on MM cell survival. However, apoptosis assays using annexin-V and propidium iodide staining indicated that Eos SN did not significantly enhance HMCL survival (data not shown).

Although our results thus far indicate that Eos can induce malignant PC proliferation through soluble factors, it does not rule out the possibility that a component which is contact-dependent may also exist. To formally test the hypothesis that Eos can enhance malignant PC proliferation in a contact-dependent manner, we compared the proliferation of HMCLs either alone, in direct contact with Eos, or in the presence of Eos that are separated by a transwell. We predicted that if a contact-dependent component exists, the HMCLs in direct co-culture with the Eos would show an enhanced proliferation over the ones in co-culture through a transwell. Our data revealed that although the Eos-induced MM proliferation tended to be greater when the cells were co-cultured in direct contact as compared to across transwells, the difference was not statistically significant in 2 of the 3 HMCLs tested (Figure 3C). Because these data suggest that Eos-mediated enhancement of MM cell proliferation is largely soluble in nature, our remaining studies focused on achieving a better understanding of the contact-independent aspect of Eos-induced MM proliferation.

### Proliferation of primary CD138^+^ MM cells is enhanced by treatment with Eos culture supernatant

**Figure 4:**
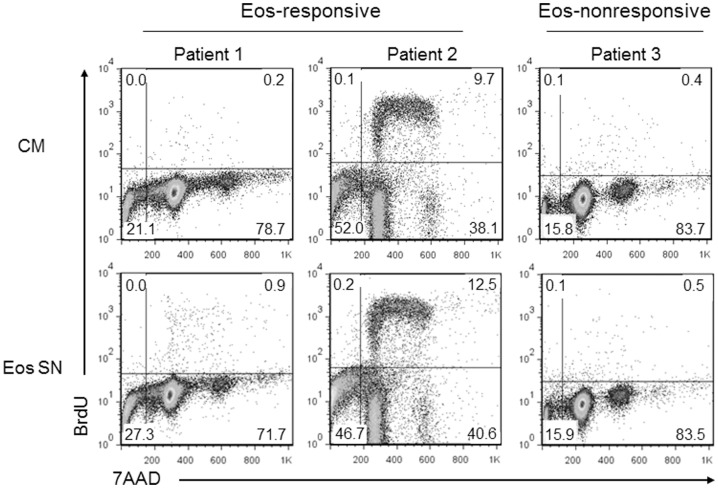
Proliferation of primary CD138+ MM cells is enhanced by treatment with Eos SN. Proliferation of the CD138+ cells upon treatment with Eos SN or CM was assessed by BrdU labeling. The figure shows representative flow cytometric analysis of the BrdU staining of 2 Eos-responsive (Patient 1 and 2) and 1 Eos-nonresponsive (Patient 3) samples. A total of 6 samples were analyzed.

So far, our findings regarding the proliferation-inducing effect of Eos on malignant PCs have been based solely on HMCLs. To assess the relevance of our results to non-cell line systems, we isolated primary CD138^+^ cells from BM of 6 MM patients and tested their proliferation upon treatment with BM Eos culture supernatant. We observed an increased percentage of primary CD138^+^ MM cells that became BrdU^+^ upon treatment with Eos SN compared to those that were cultured in control media in 4 out of 6 primary samples tested (Figure 4 and Table 1). Our findings that proliferation of some, but not all, primary MM cells can be enhanced by Eos mirror that which was observed with our MM cell lines.

**Table 1:** Proliferation of primary CD138+ MM cells.

Eos-responsive			Eos-nonresponsive		
	% S phase			% S phase	
Patient #	CM	Eos SN	Patient #	CM	Eos SN
1	0.2	0.9	3	0.4	0.5
2	9.7	12.5	6	1.6	1.6
4	0.1	0.4			
5	0.5	0.8			

CM, control media; Eos y CD138+ MM cellstter with a color that makes the arrow stand out even). Anyway, I have a new MM image no.

### BM Eos and SCs exhibit non-redundant roles in their support of MM cell proliferation

**Figure 5:**
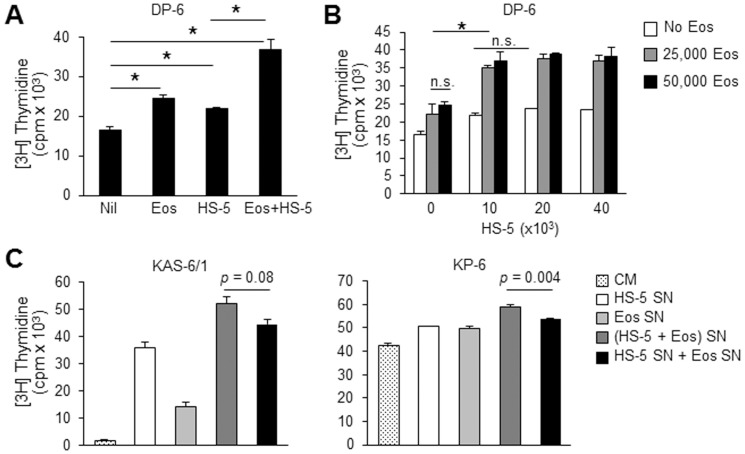
BM Eos and stromal cells (SC) have non-redundant roles in their support of MM cell proliferation. (**A**) DP-6 cells were cultured either alone, in the presence of BM Eos, partially irradiated HS-5, or both. Cells were cultured at a ratio of 1∶1∶0.4 (DP-6:Eos:HS-5). Proliferation of DP-6 was assessed using a [3H]TdR-incorporation assay. (**B**) Varying numbers of BM Eos and HS-5 cells were co-cultured with 50,000 DP-6 cells and the proliferation of these cells was evaluated using [3H]TdR-incorporation. (**C**) Proliferation of KAS-6/1 (left) and KP-6 (right) was assessed after treatment with culture SN from HS-5, Eos, or HS-5 and Eos co-cultures. Additionally, HS-5 SN and Eos SN were mixed at a 1∶1 ratio and used to treat HMCLs. Results are representative of 3 independent experiments. * *p*<0.05.

A model of a “multi-component PC niche” has recently been proposed whereby CXCL12^+^ BM SCs provide scaffolding and basic survival factors for the PCs and a second support cell type in the niche further contributes to the longevity of PCs through additional soluble factors.[22] Thus, we next attempted to define the role of Eos in a more complex MM cell niche that includes BM SCs. Proliferation of DP-6 cells was assessed in co-culture either with BM Eos, with HS-5 stromal cells, or with both BM Eos and HS-5. As shown in Figure 5A, co-culture of DP-6 with BM Eos and HS-5 cells independently enhanced DP-6 proliferation. Moreover, when the three cell types were cultured together, the proliferation of DP-6 cells was increased to an even greater degree. Co-cultures using SCs derived from BM of MM patients and normal healthy donors also showed similar results (data not shown). We reasoned that this finding could be explained by one of two possibilities: 1) BM Eos and HS-5 produce the same panel of pro-growth cytokines, and therefore the augmented proliferative response to these two cell types together is due to an increase in the concentrations of these cytokines; or 2) BM Eos and HS-5 produce different pro-growth cytokines to independently increase MM cell proliferation by their signaling through distinct molecular pathways. To determine which of these possibilities might best explain our observations in Figure 5A, we co-cultured DP-6 cells with varying numbers of BM Eos and HS-5 cells. If Eos and HS-5 are producing the same cytokines, we would expect that the absence of Eos can be rescued by the addition of greater numbers of HS-5 cells and vice versa. However, our data showed the contrary and revealed that increasing numbers of Eos or HS-5 alone could not induce the same level of DP-6 proliferation as when both Eos and HS-5 cells were present (Figure 5B). Taken together, our results suggest that BM Eos and SCs have non-redundant roles in their support of MM cell proliferation.

### Eos and SCs interact to provide optimal support for MM cell proliferation

Next, we questioned whether crosstalk might exist between the Eos and SCs such that their ability to induce MM cell proliferation is enhanced by the co-culture. To test the hypothesis that Eos and SCs interact to result in secretion of more and/or different proliferation-inducing factors for MM cells, we collected culture SNs from Eos alone, HS-5 alone, or Eos and HS-5 co-cultures and assessed proliferation of KAS-6/1, DP-6, and KP-6 upon treatment with these SNs. Specifically we compared the proliferation of these HMCLs when treated with SN from the Eos and HS-5 co-cultures to when treated with the combined SNs from the Eos cultures and the HS-5 cultures. Our results showed that while the combined SN from the individual cultures induced proliferation of KAS-6/1, DP-6, and KP-6 more than either SNs did alone, the SN collected from the Eos and HS-5 co-culture induced an even greater proliferative response (Figure 5C and data not shown). These data are suggestive that crosstalk may exist between Eos and HS-5 to result in an optimal microenvironment for MM cell growth.

### A soluble factor(s), and not microparticles, mediates Eos-induced HMCL proliferation

**Figure 6:**
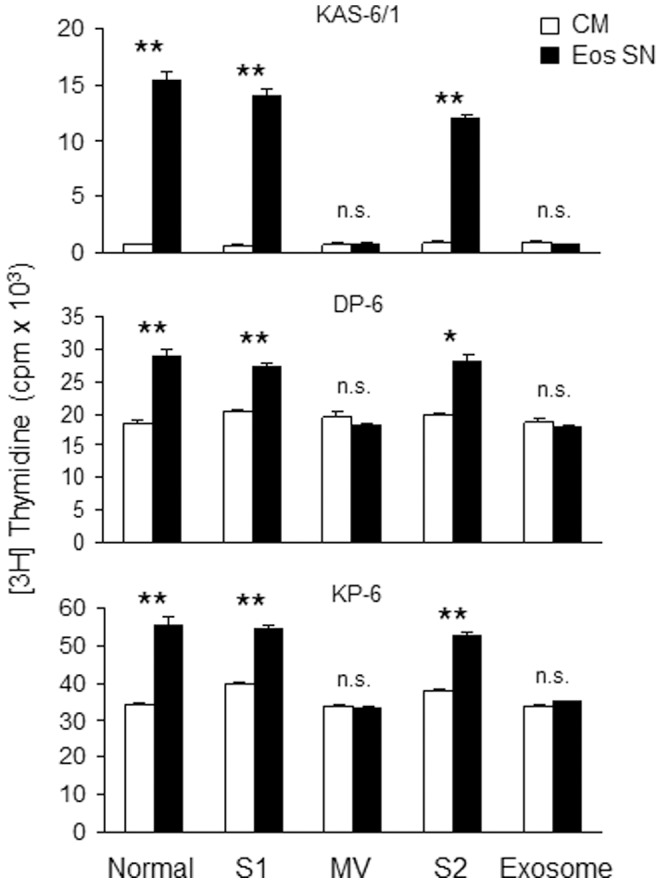
Eos secrete a soluble factor(s) to enhance HMCL proliferation. MV and exosomes were isolated from BM Eos SN. S1 and S2 are the MV-free and exosome-free supernatants, respectively. The various fractions were tested for their ability to stimulate HMCL proliferation as assessed by [3H]TdR-incorporation assay. Results are representative of 3 independent experiments. * *p*<0.01; ** *p*<0.001; n.s., not significant.

Finally, we began to investigate the mechanism by which Eos enhance HMCL proliferation. As BM SCs have been shown to modulate MM cell growth via release of miRNA-containing exosomes,[6], [23] we first evaluated whether Eos-derived microparticles, including MVs and exosomes, could be mediating the increase in MM proliferation. We utilized ultracentrifugation techniques to isolate MVs and exosomes in the Eos SN and demonstrated that the growth-stimulating effect of Eos SN was the result of a soluble factor(s) and could not be attributed to possible MVs and exosomes shed by Eos (Figure 6).

### Eos support HMCL proliferation through a mechanism(s) independent of APRIL and IL-6

**Figure 7:**
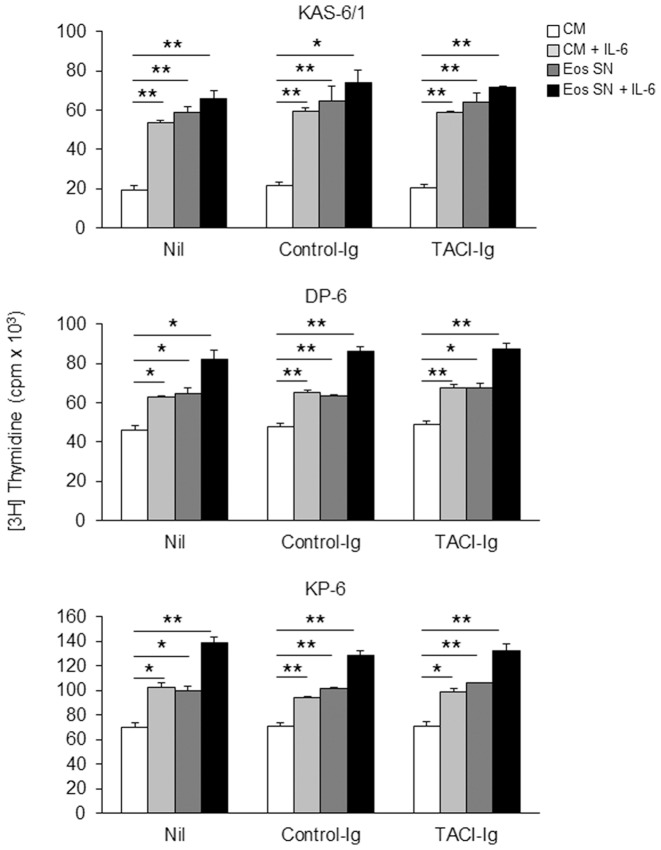
Eos support HMCL proliferation via a mechanism(s) independent of APRIL. KAS-6/1 (top), DP-6 (middle), and KP-6 (bottom) cells were cultured in CM or Eos SN ±1 ng/ml IL-6 or in the presence of 2 µg/ml TACI-Ig or control-Ig. Proliferation was assessed by [3H]TdR-incorporation assay. Results are representative of 3 independent experiments. * *p*<0.01; ** *p*<0.001.

Prior reports in the mouse indicate that Eos can support normal long-lived PC survival in the BM via their secretion of IL-6 and APRIL [13]. Given that our HMCLs exhibit little or no enhanced proliferation in the presence of recombinant APRIL (data not shown), we considered it unlikely that APRIL was the primary factor driving Eos-induced HMCL proliferation. To test this possibility directly, we cultured KAS-6/1, DP-6, and KP-6 cells in Eos SN or CM ±IL-6 and in the presence of TACI-Ig or control-Ig. Our data revealed that TACI-Ig did not abolish the proliferation-inducing effect of Eos SN (Figure 7). In experiments not shown, we verified the APRIL blocking activity of TACI-Ig using cell lines that exhibit a modest proliferative response to recombinant APRIL.

**Figure 8:**
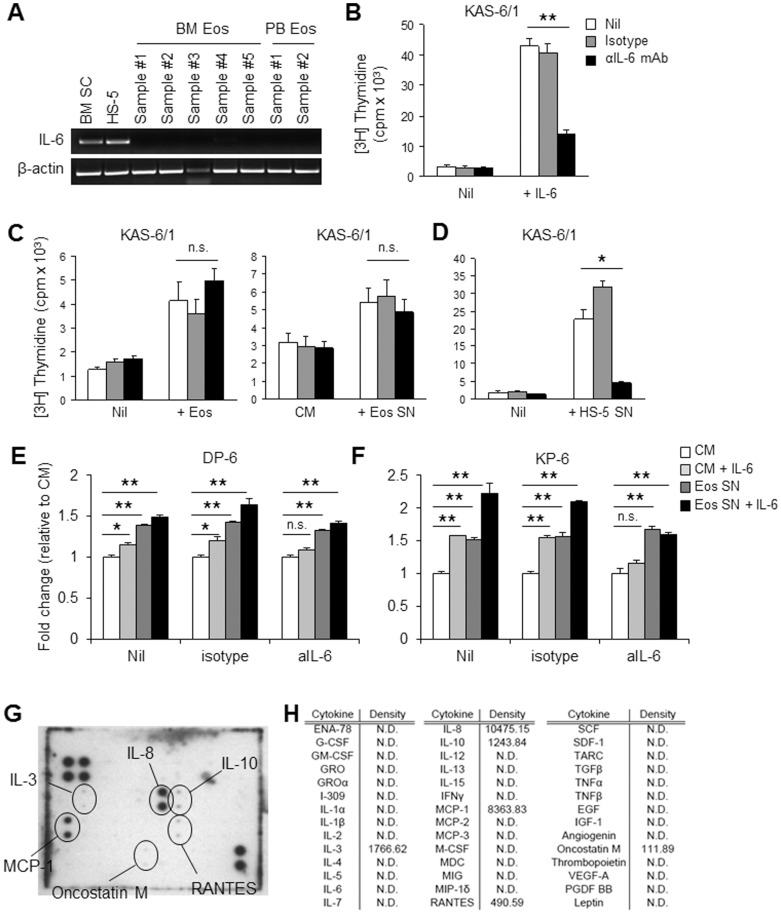
Eos support HMCL proliferation through a mechanism(s) independent of IL-6. (**A**) PCR analysis of mRNA from HS-5 cells, BM SC and Eos from MM patients, and PB Eos from healthy donors evaluating expression of IL-6. β-actin was used as loading control. (**B**) Neutralizing antibody against IL-6 (αIL-6 mAb) or isotype control antibody was added to KAS-6/1 cells cultured alone or in the presence of 1 ng/ml IL-6. (**C–D**) KAS-6/1 cells were cultured with BM Eos (**C, left**), Eos SN (**C, right**), or HS-5 SN (**D**) in the presence of αIL-6 mAb or isotype control antibody. Proliferation was assessed by [3H]TdR-incorporation assay. (**E–F**) DP-6 (**E**) and KP-6 (**F**) cells were cultured in CM or Eos SN ±1 ng/ml IL-6 or in the presence of αIL-6 mAb or isotype control antibody. Proliferation was assessed by [3H]TdR-incorporation assay and the fold change relative to CM was calculated for each group. (**G**) Eos SN was evaluated for the presence of various cytokines using a membrane array containing antibodies to 42 different human cytokines. (**H**) Volumetric analysis of the membrane array in (**G**). [3H]TdR-incorporation and cytokine array data are representative of 3 independent experiments. * *p*<0.05; ** *p*<0.001; n.s., not significant; N.D., not detected.

Initial observations that the proliferation of JMW, ALMC-2, and ANBL-6 (3 of our IL-6-responsive HMCLs) was not induced by co-culture with Eos (Figure 2) provided the first clues to suggest that human Eos exert their effects on malignant PCs independent of IL-6. Furthermore, the ability of Eos to increase KAS-6/1, DP-6, and KP-6 proliferation at already saturating levels of IL-6 provided yet another piece of evidence toward a mechanism distinct from IL-6 to explain the Eos-induced HMCL proliferation. To formally test this hypothesis, we first examined the expression of IL-6 mRNA by Eos and SCs and found that while BM SCs from MM patients and the HS-5 stromal cells expressed IL-6 mRNA, this expression was absent in both human BM and PB Eos (Figure 8A). As Eos are known to store many pre-formed proteins in their secondary granules to be readily released,[24] we wanted to ensure that the absence of IL-6 transcripts corresponded to the lack of IL-6 protein in human Eos. In neutralization studies we demonstrated that an anti-IL-6 mAb was efficient at neutralization of human recombinant IL-6 but had little effect on the induction of KAS-6/1 proliferation by treatment with Eos SN or by co-culture with Eos (Figure 8B and 8C). In contrast, we showed that the proliferation-inducing effect of HS-5 cells is abolished in the presence of neutralizing anti-IL-6 mAb (Figure 8D), confirming that BM SCs promote malignant PC proliferation through the secretion of IL-6. Similar to our findings with KAS-6/1, neutralization studies with DP-6 and KP-6 demonstrated that the IL-6-induced proliferation, but not the Eos-induced proliferation, is abolished by anti-IL-6 mAb (Figure 8E and 8F). Lastly, to begin to identify the mechanism(s) through which Eos support malignant PC proliferation, we tested our Eos culture supernatant for the presence of various cytokines using membrane arrays containing antibodies to 42 different human cytokines. The array was first verified to have minimal cross-reactivity with bovine serum proteins (Figure S2). Subsequently, these arrays identified several candidate cytokines that are produced by human Eos, including IL-8, MCP-1, IL-10, RANTES, oncostatin M, and IL-3 (Figure 8G and 8H). However, these recombinant cytokines have thus far failed to stimulate the proliferation of KAS-6/1, DP-6, and KP-6 MM cell lines (data not shown).

## Discussion

Normal and malignant PCs rely on their niche within the BM for survival and proliferation, respectively.[5], [9], [25], [26] We have identified a novel component of this niche – the eosinophils – that contributes to the enhanced proliferation of MM cells. Our data revealed the frequent proximity of eosinophils and PCs in the BM of MM patients as well as normal donors. Additionally, we provide evidence for the biological significance of this colocalization as the proliferation of MM cells is enhanced in the presence of Eos when cultured *in vitro*. The MM cell proliferation induced by Eos was further shown to be mechanistically distinct from that induced by BM SCs.

Quantitative analysis of the BM biopsies from normal donor and patients with different stages of monoclonal gammopathies demonstrated an increase in the percentage of Eos that are in close proximity with PCs with disease progression. These findings may suggest that Eos that are utilized by the malignant PCs are selectively retained in the BM while other cells are crowded out of the BM during clonal expansion of the malignant cells. Alternatively, it is possible that MM cells may secrete chemotactic factors to actively recruit Eos to the tumor niche in order to gain a proliferative advantage. Additional studies are needed to discriminate between these possibilities. We also observed that approximately 50% of the Eos in BM from normal donors are in close proximity to PCs. This begs the question whether Eos have any impact on normal PC biology in humans. However, our current study does not provide information regarding the biological significance of this colocalization nor the mechanism of this colocalization. Chu et al (2011) suggested that, at least in the mouse, Eos and PCs may localize together within the BM via their coordinated migration toward a common source of CXCL12. It is possible that this holds true in humans as well, however the exact mechanism as well as the consequence of this colocalization remains unknown at this time and warrants further investigation.

Results from our study indicate that the effects of Eos on MM cell proliferation are largely contact-independent. Although we did observe a trend toward a greater induction of proliferation when MM cells were co-cultured in direct contact with Eos as compared to across transwells, the difference was not statistically significant. To further explore the contact-independent induction of MM proliferation by Eos, we first tested whether the active soluble Eos-derived molecule(s) was IL-6 and/or APRIL, as each has been shown to be produced by many other cell types within the BM PC niche and to support the survival and proliferation of normal and malignant PCs, respectively.[6], [8], [11], [27], [28] However, our results indicated that the growth-promoting activity of Eos supernatants is not a result of IL-6 or APRIL prompting us to consider other soluble molecules. Use of cytokine arrays allowed us to identify other candidate cytokines in the Eos culture SN, including IL-8, MCP-1, IL-10, RANTES, oncostatin M, and IL-3. *In vitro* stimulation of HMCLs with these recombinant cytokines did not induce significant proliferation (results not shown). These studies suggest a yet-to-be identified factor is instead produced by Eos, however, it also remains possible that Eos-induced MM cell proliferation depends upon a precise combination of these cytokines.

Our observations that the effects of Eos on MM cell proliferation are independent of IL-6 and APRIL signaling raise an interesting question regarding differences between humans and mice. In a prior report which shows the requirement of Eos for the maintenance of long-lived humoral responses in mice, IL-6 and APRIL were demonstrated to be the major factors produced by Eos to influence the survival of BM PCs. [13] Although the goal of our current study was to evaluate the biological significance of Eos in the malignant PC niche, our results have implications for the normal PC niche as well. Thus, the evident lack of IL-6 production and secretion by human Eos suggests that in humans Eos are either not necessary for the long term survival of BM PCs or are utilizing an IL-6-independent signaling pathway to influence PC longevity. Additionally, unlike the findings by Chu et al that in mice BM Eos can enhance normal PC survival, our data indicate that human Eos influence MM cell proliferation without affecting cell survival. Whether this discrepancy reflects a difference in mouse compared to humans or a difference between normal and malignant PCs remains unknown.

We also obtained evidence that Eos SN could increase the fraction of some primary MM cells in S phase. Clinically, the plasma cell labeling index (PCLI) is used to prognosticate patients with MM. PLCI relies on the use of BrdU to measure the percent of malignant cells that are actively undergoing DNA synthesis (i.e., in S phase of the cell cycle).[29], [30] As MM is a low proliferative disease, an elevation of the PCLI to greater than 1% is highly significant as it is suggestive of early disease progression and is associated with poor prognosis.[31], [32] We used this method to assess proliferation of primary MM cells in the presence or absence of Eos. Once again, although the baseline rate of proliferation of these cells was very low, the ability of Eos to as much as quadruple the percent of BrdU positive cells in our assays of primary MM patient samples may suggest clinical relevance and significance.

We note that of the 6 HMCLs examined, only 3 cell lines showed enhanced proliferation in the presence of Eos, namely KAS-6/1, DP-6, and KP-6. Likewise, we observed a similar phenomenon with primary MM samples where CD138+ BM cells from 4 out of 6 MM patients had an increased BrdU uptake upon culture in Eos SN. In an attempt to elucidate the mechanism underlying this Eos-induced MM cell proliferation, we compared our Eos-responsive cell lines to the Eos-nonresponsive cell lines. We found that an individual cell line's responsiveness to Eos could not be predicted by cytogenetic abnormalities such as hyperdiploidy, chromosomal translocations, or gene-specific mutations. It is also possible that the expression levels of specific receptors or signaling molecules may differ between the cell lines to explain the presence or absence of response to Eos. Gene expression profiling data from these cell lines identified a number of genes whose expression is upregulated in Eos-responsive cell lines (Table S2), however, the biological significance of these overexpressed genes and their relevance to the responsiveness toward Eos have not yet been explored and are currently under investigation.

While our current study focused on the role of Eos in MM only as it pertains to the induction of MM cell proliferation, Eos may in fact also influence the biology of the disease in other ways. For example, one characteristic clinical finding in MM patients is the presence of osteolytic bone lesions. These lesions are the result of an imbalance between osteoblast and osteoclast activities wherein bone resorption by osteoclasts predominates.[33], [34] MCP-1, produced by MM cells through p38 signaling, has been shown to result in increased RANK expression by osteoclasts promoting their bone-destruction activity. [35] Elevated levels of IL-3 have been observed in BM plasma obtained from MM patients compared to that obtained from healthy controls.[36] IL-3 was demonstrated to be produced by MM cells as a consequence of deregulated expression of AML-1 class transcription factors, and functionally it induces osteoclast formation as well as inhibit osteoblast differentiation. [36], [37] Although we have no evidence that Eos can influence the expression of MCP-1 or IL-3 by MM cells, it is interesting to note that both MCP-1 and IL-3 were detected in our Eos culture SN, suggesting that Eos may in fact participate in the osteolytic bone disease in MM.

The role of Eos in other cancers has been previously investigated. While Eos appear to have a pro-tumor growth effect in some malignancies, in others they are associated with good prognosis having an anti-tumor effect.[38] With respect to lymphoproliferative disorders (LPD), hypereosinophilia has been not uncommonly found in both malignant T-cell LPD (e.g., mycosis fungoides/Sezary syndrome, angioimmunoblastic T-cell leukemia, and adult T-cell leukemia/lymphoma) and malignant B-cell LPD (e.g., classical Hodgkin lymphoma and B-cell acute lymphoblastic leukemia).[39] The prognostic impact of hypereosinophilia in these diseases has mostly either been negative or uncharacterized. To date, much of this poor prognosis associated with hypereosinophilia has been attributed to Eos-mediated organ damage and complications as well as a reflection on Th1/Th2 imbalance leading to improper anti-tumor immunity, however, the direct impact of Eos on the biology of the malignant cells in these diseases have not yet been examined. Results from our current study suggest that this possibility warrants further investigation.

Although elevated numbers of Eos have not been consistently reported in MM patients and therefore cannot be used as a prognostic factor for the disease, the results of our current study indicate that Eos may in fact contribute to the biology and pathology of MM. Of interest, a MM patient who had coincident eosinophilia has been described in a case report.[40] The use of chemotherapy along with steroids, to which Eos are particularly sensitive, led to the successful reduction of both Eos and PCs in the BM indicating possible biological interactions between the two cell types. As our data suggest a novel, unidentified mechanism for the induction of MM cell proliferation by Eos which is independent of IL-6 and APRIL, we believe that the targeting of these cells in the BM microenvironment may be efficacious in the treatment of MM.

## Supporting Information

Figure S1 **Proliferation of HMCL is not affected by IL-5.** HMCL were cultured in the presence of 1 ng/ml IL-5, 1 ng/ml IL-6, or both. Proliferation of HMCL was assessed at day 3 of culture by [3H]TdR-incorporation. Results are representative of 3 independent experiments. * p<0.05; ** p<0.001; n.s., not significant.
(DOCX)

Figure S2 **Minimal cross-reactivity of fetal bovine serum proteins to human cytokine array.** IMDM containing 10% FCS and 1 ng/ml recombinant human IL-5 was used to simultaneously test cross-reactivity of the array to bovine serum as well as its sensitivity for cytokine detection.
(DOCX)

Table S1 **Quantitative analysis of Eos in BM biopsies from normal donors and from patients with monoclonal gammopathy.**
(DOCX)

Table S2 **Genes overexpressed in Eos-responsive MM cell lines compared to in Eos-nonresponsive cell lines based on gene expression profiling data.**
(DOCX)
