# Supplementary material for: Induction of Malignant Plasma Cell Proliferation by Eosinophils
Source: PLoS One. 2013 Jul 22;8(7):e70554. doi: 10.1371/journal.pone.0070554 (PMC3718740; doi:10.1371/journal.pone.0070554)
Supplement: Figure S1 — Proliferation of HMCL is not affected by IL-5. HMCL were cultured in the presence of 1 ng/ml IL-5, 1 ng/ml IL-6, or both. Proliferation of HMCL was assessed at day 3 of culture by [3H]TdR-incorporation. Results are representative of 3 independent experiments. * p<0.05; ** p<0.001; n.s., not significant. (DOCX) [file pone.0070554.s001.docx]

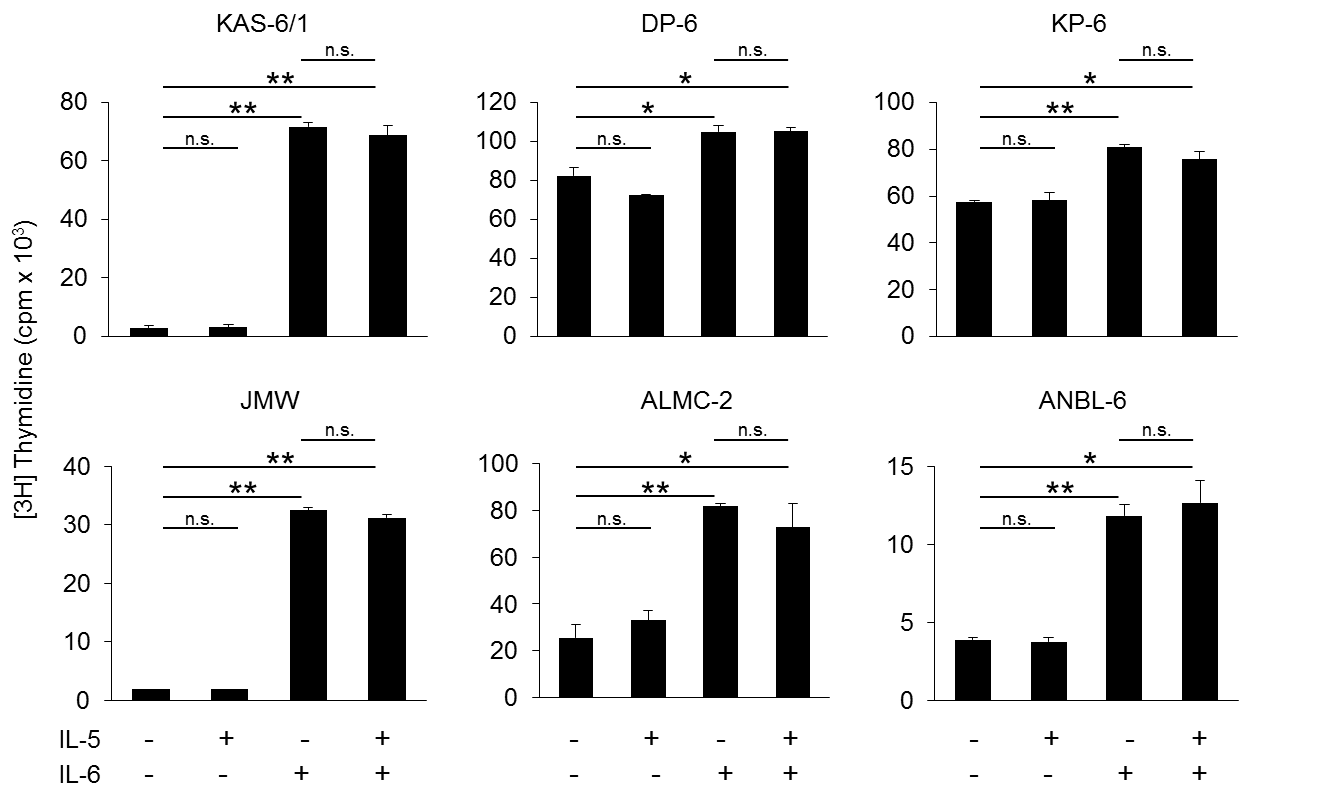


**Figure S1. Proliferation of HMCL is not affected by IL-5.** HMCL were cultured in the presence of 1 ng/ml IL-5, 1 ng/ml IL-6, or both. Proliferation of HMCL was assessed at day 3 of culture by [3H]TdR-incorporation. Results are representative of 3 independent experiments. * *p* < 0.05; ** *p* < 0.001; n.s., not significant.
