# Supplementary material for: Induction of Malignant Plasma Cell Proliferation by Eosinophils
Source: PLoS One. 2013 Jul 22;8(7):e70554. doi: 10.1371/journal.pone.0070554 (PMC3718740; doi:10.1371/journal.pone.0070554)
Supplement: Figure S2 — Minimal cross-reactivity of fetal bovine serum proteins to human cytokine array. IMDM containing 10% FCS and 1 ng/ml recombinant human IL-5 was used to simultaneously test cross-reactivity of the array to bovine serum as well as its sensitivity for cytokine detection. (DOCX) [file pone.0070554.s002.docx]

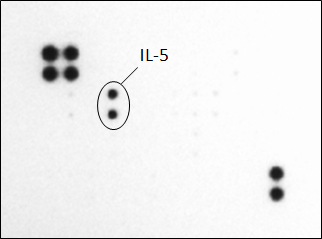


**Figure S2. Minimal cross-reactivity of fetal bovine serum proteins to human cytokine array.** IMDM containing 10% FCS and 1 ng/ml recombinant human IL-5 was used to simultaneously test cross-reactivity of the array to bovine serum as well as its sensitivity for cytokine detection.
