# Supplementary material for: Induction of Malignant Plasma Cell Proliferation by Eosinophils
Source: PLoS One. 2013 Jul 22;8(7):e70554. doi: 10.1371/journal.pone.0070554 (PMC3718740; doi:10.1371/journal.pone.0070554)
Supplement: Table S1 — Quantitative analysis of Eos in BM biopsies from normal donors and from patients with monoclonal gammopathy. (DOCX) [file pone.0070554.s003.docx]

**Table S1. Quantitative analysis of Eos in BM biopsies from normal donors and from patients with monoclonal gammopathy.**

| Sample # | Diagnosis | % PC | Total # Eos contacting PC | Total # Eos within 3-cell distance of PC | Total # Eos not near PC |
| --- | --- | --- | --- | --- | --- |
| 1 | ND | <1 | 23 | 46 | 51 |
| 2 | ND | <1 | 25 | 53 | 50 |
| 3 | ND | <1 | 39 | 56 | 67 |
| 4 | ND | <1 | 16 | 20 | 34 |
| 5 | ND | <1 | 36 | 51 | 70 |
| 6 | MGUS | 3 | 14 | 50 | 51 |
| 7 | MGUS | 6 | 48 | 52 | 96 |
| 8 | MGUS | 9 | 32 | 42 | 12 |
| 9 | MM | 12 | 33 | 14 | 8 |
| 10 | SMM | 14 | 58 | 55 | 14 |
| 11 | SMM | 22 | 27 | 3 | 0 |
| 12 | SMM | 28 | 29 | 9 | 1 |
| 13 | MM | 35 | 76 | 22 | 12 |
| 14 | MM | 45 | 141 | 88 | 30 |
| 15 | MM | 82 | 39 | 8 | 5 |

*Counts reflect sum of the number of Eos across 6 medium-power (40x) fields.*

*Diagnosis and % PC were obtained from patient clinical records.*
