# Supplementary material for: Induction of Malignant Plasma Cell Proliferation by Eosinophils
Source: PLoS One. 2013 Jul 22;8(7):e70554. doi: 10.1371/journal.pone.0070554 (PMC3718740; doi:10.1371/journal.pone.0070554)
Supplement: Table S2 — Genes overexpressed in Eos-responsive MM cell lines compared to in Eos-nonresponsive cell lines based on gene expression profiling data. (DOCX) [file pone.0070554.s004.docx]

**Table S2. Genes overexpressed in Eos-responsive MM cell lines compared to in Eos-nonresponsive cell lines based on gene expression profiling data.**

| Gene Symbol | Gene Title | ALMC-2 | ANBL-6 | JMW | **Average (Eos-nonresponsive)** | KAS-6/1 | KP-6 | DP-6 | **Average (Eos-responsive)** | **Eos-responsive / Eos-nonresponsive** |
| --- | --- | --- | --- | --- | --- | --- | --- | --- | --- | --- |
| S100A4 | S100 calcium binding protein A4 | 132.5 | 132.9 | 133.1 | **132.9** | 831.0 | 1788.5 | 4423.8 | **2347.7** | **17.7** |
| CD9 | CD9 molecule | 334.9 | 1168.0 | 321.4 | **608.1** | 3598.9 | 4268.8 | 5980.8 | **4616.2** | **7.6** |
| MUC1 | mucin 1, cell surface associated | 186.1 | 221.9 | 563.1 | **323.7** | 3581.2 | 747.2 | 2906.5 | **2411.6** | **7.5** |
| MUC1 | mucin 1, cell surface associated | 125.8 | 104.3 | 193.8 | **141.3** | 1287.4 | 346.4 | 1143.8 | **925.9** | **6.6** |
| TIMP1 | TIMP metallopeptidase inhibitor 1 | 118.9 | 240.5 | 558.7 | **306.0** | 1576.3 | 2137.6 | 1456.0 | **1723.3** | **5.6** |
| ANXA1 | annexin A1 | 95.3 | 110.5 | 186.8 | **130.8** | 601.1 | 625.2 | 917.4 | **714.5** | **5.5** |
| PXDN | peroxidasin homolog (Drosophila) | 127.0 | 121.6 | 1709.1 | **652.5** | 830.4 | 5294.8 | 4221.3 | **3448.8** | **5.3** |

*Values in table represent relative signal strength.*
